# Transcatheter Aortic Valve Replacement Versus Surgical Aortic Valve Replacement in Bicuspid Aortic Valve Stenosis—We Need a Well-Designed Randomized Control Trial

**DOI:** 10.3390/jcm13216565

**Published:** 2024-10-31

**Authors:** Kendra J. Grubb, Stephanie K. Tom, Joe Xie, Kanika Kalra, Anton Camaj

**Affiliations:** 1 Division of Cardiothoracic Surgery, School of Medicine, Emory University, Atlanta, GA 30308, USA; kanika.kalra@emory.edu; 2 Department of Surgery, Emory University School of Medicine, Atlanta, GA 30308, USA; stephanie.tom@emory.edu; 3 Division of Cardiology, Department of Medicine, Emory University School of Medicine, Atlanta, GA 30308, USA; joe.xie@emory.edu (J.X.); anton.camaj@emory.edu (A.C.)

**Keywords:** TAVR, SAVR, bicuspid aortic stenosis, randomized controlled trial

## Abstract

Bicuspid aortic stenosis is a common pathology, typically seen in patients a decade younger than those with tricuspid valves. Surgical aortic valve replacement has been the mainstay treatment for bicuspid disease, especially considering the prevalence of concomitant aortic aneurysmal pathology. Transcatheter aortic valve replacement has shown equivalent results in bicuspid compared to tricuspid pathology in highly selected patient populations in single-arm registries and observational studies. For older patients with favorable bicuspid pathology, TAVR is reasonable. However, as younger patients with longer life expectancy are now being treated with TAVR, what is “best” is a question only answered by a well-designed randomized controlled trial. Herein, we describe the current evidence for treating bicuspid aortic stenosis and provide a framework for future trials. Yet, the question of equipoise remains, and who will we enroll?

## 1. Introduction

Bicuspid aortic valve (BAV) is the most common congenital heart valve anomaly affecting approximately 1–2% of the population [1]. A BAV is characterized by having only two cusps and well-formed leaflets instead of three and portends an increased risk of degenerative aortic stenosis (AS) in adulthood, classically requiring intervention a decade earlier than trileaflet patients.

While these valves have traditionally been treated surgically via either repair (if regurgitant) or replacement techniques, with advancements in medical technology, transcatheter aortic valve replacement (TAVR) has emerged as an alternative to surgical aortic valve replacement (SAVR) for treating symptomatic severe AS across all-risk groups, with results comparable to surgery for tricuspid disease [2,3,4,5,6,7]. However, the optimal treatment strategy for patients with BAV AS remains unknown as these patients were excluded from landmark randomized controlled trials (RCT) comparing TAVR and SAVR.

### 1.1. Anatomy

**Figure 1:**
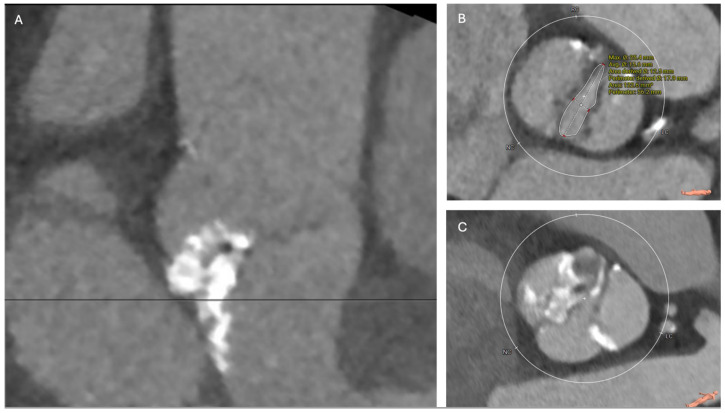
Bicuspid Aortic Valve Stenosis with High-Risk Features. Examples of bicuspid aortic stenosis pathology with increased complication risk due to severe calcification or abnormal valve morphology. (**A**) Sievers 1 bicuspid with severe calcification of the aortic valve leaflets extending into the left ventricular outflow tract, calcium score 11,017, (**B**) Sievers 0 bicuspid with large annulus sizing for the largest TAVR valves (perimeter 89.5mm and aortic valve area 634 mm^2^), with an elliptical orifice (25.4 mm × 15.6 mm) at the level of the leaflets, making valve selection difficult, (**C**) Sievers 0 bicuspid with a large annulus (perimeter 90.3 mm and area 637.4 mm^2^), dilated sinus of Valsalva measuring 45 mm, and heavy calcium and abnormal morphology at the leaflet level.

There are various classifications of BAV, but the most widespread is the Sievers classification [8]. Sievers phenotypes are categorized according to the number (0, 1, or 2) of fusions between adjacent leaflets, known as raphe. Sievers type 1 is the most common phenotype, characterized by fusion of the left and right coronary cusp and leaflets in most patients (70%) [8]. Bicuspid AS poses unique anatomic challenges compared with tricuspid AS. Stenotic BAVs typically have large asymmetric sinuses and heavily calcified leaflets accompanied by raphe, with calcium typically extending into the left ventricular outflow tract, exposing patients to the risk of suboptimal TAVR expansion, paravalvular leak, annular rupture, and conduction disturbances (Figure 1) [9].

Additionally, more than a quarter of patients with BAV AS have an associated aortopathy characterized by dilation of the aortic valve annulus, aortic root, or ascending aorta that TAVR cannot treat [1,10]. The prevalence of BAV-associated aortopathy ranges widely between 20% and 84%, depending on the imaging technique and aortic size thresholds used to define aortic dilation [11]. Accordingly, the risk of aortic events during TAVR (e.g., aortic injury or rupture) conveyed by concomitant aortopathy may be as high as 8-fold relative to the general population [12]. Moreover, BAV patients typically have an aorta with a more horizontal orientation, making transcatheter valve delivery and positioning more challenging.

### 1.2. Current Guideline Recommendations

The most recent 2020 American College of Cardiology/American Heart Association (ACC/AHA) guideline for managing valvular heart disease gives TAVR a class 2b indication as an alternative to SAVR in BAV patients with symptomatic severe stenosis and life expectancy of less than 20 years (65 and older) [13]. Additionally, while the ACC/AHA guideline offers recommendations for the surgical management of BAV AS in the setting of concomitant aortopathy, there is no recommendation for transcatheter treatment options in these more complex anatomies or in guidelines specific to aortic disease [14,15,16]. The 2021 European Society of Cardiology and the European Association for Cardio-Thoracic Surgery guidelines do not provide formal recommendations for TAVR in patients with BAV. Instead, the guidance states *SAVR remains more appropriate in patients with AS affecting a bicuspid valve and in those with associated disease (e.g., aortic root dilatation, complex coronary disease, or severe mitral regurgitation) requiring a surgical approach*. The European guidelines also use an age cut-off of 75 years for TAVR, reserving surgery for low-risk patients under 75 [17].

With the prevalence of concomitant aortic dilation, the guidelines recommend operative replacement (class 2a) with an aortic root ≥ 5.5 cm or dilation ≥ 5.0 cm with additional risk factors, including bicuspid anatomy (class 2a) [13,17]. Aortic root or ascending aorta replacement is warranted at ≥4.5 cm in patients with an indication for aortic valve surgery. Table 1 compares the 2020 ACC/AHA and 2021 ESC/EACTS guidelines related to BAV with or without concomitant aortic root disease.

### 1.3. Surgical Aortic Valve Replacement for Bicuspid Aortic Valve Stenosis

Surgical outcomes in BAV AS patients, who are more frequently younger and less comorbid than tricuspid AS patients, are excellent, with low in-hospital mortality rates ranging from 0.9% to 2.4% [18,19]. Long-term survival following SAVR is favorable as well. In a series of 231 patients who underwent SAVR with concomitant ascending aortic replacement, the 10-year survival rate was 88% [19]. Similarly, in another study, the 10- and 15-year survival rates were 59–83% and 37–70%, respectively, following SAVR with or without concomitant aortic dilation and intervention [18]. Furthermore, these results extend to patients with BAV AS and aortic dilation undergoing isolated SAVR for 15 years of follow-up [20].

To that end, deciding when to perform concomitant aortic root or ascending surgery is nuanced. The risk of a more extensive operation must be weighed against the risk of future adverse aortic events. Kim et al. observed a progression of aortic dilation after isolated SAVR in BAV patients, but not in tricuspid valve patients [21]. Although the threshold for concomitant aortic root replacement at the time of SAVR for BAV disease is 4.5 cm, studies have shown that patients with BAV disease and aortic dilation between 4.0 cm and 5.0 cm who underwent isolated SAVR were at low risk of adverse aortic events at their 15-year follow-up [22]. Much of this risk depends on the patient’s age and longevity at the time of the operation, and those with longer life expectancy have enough years for the aortopathy to become a concern. Overall, surgery provides excellent procedural and long-term outcomes in young BAV AS patients and thus far has been considered the standard of care.

### 1.4. Transcatheter Aortic Valve Replacement for Bicuspid Aortic Valve Stenosis

In early published series, patient selection included patients who were high-risk or inoperable, older without long life expectancy, and in whom ascending aortic replacement was not an option. First- and second-generation balloon expandable (BE) and self-expanding (SE) valves were associated with high periprocedural complications, including significant paravalvular leak (13–34%), permanent pacemaker implantation (13–43%), and 1-year mortality (4–18%) [23,24]. Moreover, patients who underwent TAVR for BAV AS had an increased risk of paravalvular leak with older generation valves due to suboptimal valve expansion in an orifice with two commissures and heavy, irregular calcium [25,26]. In the early series, aortic root rupture was a significant concern and a fatal complication more commonly linked to balloon-expandable valves or aggressive post-dilation of self-expanding valves. In BAV AS patients with excessive calcification, annulus/root rupture has been estimated to range from 0.9% to 4.5% [27]. Predicting which BAV AS patients will experience a complication has been challenging. Selecting a self-expanding valve with less aggressive post-dilation, undersizing a balloon-expandable valve, or using advanced artificial intelligence simulation have been proposed to mitigate these risks [28].

In addition, refinements in the latest generation of valves, including outer sealing skirts, the ability to recapture/reposition, and improved implantation techniques, have decreased the risks [29]. In a propensity-matched cohort of BAV (561) and tricuspid (4546) patients treated with early generation (Sapien XT and CoreValve) compared to newer generation (Sapien 3, Lotus, Evolut R) valves, BAV patients overall had more frequent procedural complications than those in the tricuspid cohort (conversion to surgery: 2.5% vs. 0.3%; *p* = 0.02; second valve implantation: 7.2% vs. 2.2%; *p* = 0.003; moderate or severe paravalvular leak: 15.9% vs. 10.3%; *p* = 0.03) [30]. Nevertheless, no significant differences in procedural complications between BAV and tricuspid patients were observed following the implantation of newer-generation devices. Data from the Society of Thoracic Surgeons/ACC Transcatheter Valve Therapies registry, comparing outcomes in BAV (5412) to tricuspid (165,547) AS patients, according to TAVR valve generations, revealed a slightly lower 1-year unadjusted risk of mortality in the BAV group (hazard ratio, 0.88; 95% CI 0.78–0.99) and similar stroke rates (hazard ratio, 1.14; 95% CI 0.94–1.39) [31]. Most procedures (81%) were performed with Sapien 3 and Evolut R values. Other propensity-adjusted studies comparing TAVR outcomes in patients with BAV to tricuspid aortic valves, irrespective of device generation, have shown similar in-hospital, 30-day, and 1-year outcomes [32,33].

Only four prospective registries have explored BAV AS outcomes [34,35,36,37], and the short-term outcomes have improved with the newer generation of transcatheter heart valves. However, the prospective registries include low numbers of highly selected patients. The relatively young age of presentation for BAV AS and the unclear long-term durability of biological valves in patients <65 years old further complicates treatment decisions, and thus, there is a lack of evidence of what is “best” for treatment. Larger cohorts with longer-term data are needed.

Taken together, outcomes in patients undergoing TAVR for BAV vs. tricuspid AS appear to be similar in terms of 30-day and 1-year mortality and periprocedural complications, particularly with newer generation TAVR valves, bringing to question whether TAVR data from RCTs in tricuspid AS can be extrapolated to those with BAV AS. A summary of published propensity-score matched cohorts comparing BAV to tricuspid valves following TAVR is shown in Table 2.

### 1.5. Transcatheter Valve Durability in Bicuspid Aortic Valve AS

Limited durability data exist for transcatheter aortic valves in bicuspid anatomy, with the longest follow-up extending to two years. Recommendations support an imaging-based definition of valve failure that confirms permanent structural changes to the leaflets and evidence of deterioration in valve hemodynamic function in the setting of clinical sequela, rather than historical surgical definitions of valve reintervention or death, which may underestimate the true incidence and timing of valve failure [39]. Bioprosthetic valve dysfunction, often a state preceding failure, is the presence of high trans-prosthetic gradients, which may overestimate the incidence of bioprosthetic valve failure [40].

Another area of inquiry concerning TAVR prostheses is whether TAVR valves offer a larger effective orifice area than surgical prostheses, given the lack of a sewing ring and supra-annular designs for some TAVR valves. In contrast, surgery may offer the benefit of aggressive annular calcium debridement and root enlargement or replacement, allowing for a larger surgical valve. In a small cohort of BAV AS patients who underwent TAVR (78) and SAVR (74), TAVR patients had smaller pre-procedural annular area, but the mean implanted prosthetic valve size was larger compared to SAVR [5]; however, this is a manufacturing labeling issue, but likely reflects a larger internal diameter leading to lower gradients or larger effective orifice area. Additionally, no statistically significant differences were observed between the predicted effective orifice area or predicted effective orifice area indexed to the patient body surface area or predicted moderate-to-severe patient prosthesis mismatch [5]. Similarly, in the BAVARD (Biscupid Aortic Valve Anatomy and Relationship with Devices) retrospective registry, the effective orifice area was comparable between BAV (101) and tricuspid (88) patients who underwent TAVR with second-generation prostheses [41]. However, BAV patients were more likely to have transcatheter heart valve underexpansion (11% in the BAV group) [41]. Underexpansion of the prosthesis could increase the risk of hypoattenuated leaflet thickening or thrombosis, thereby limiting valve durability. Overall, the short-term durability of transcatheter valves seems acceptable when comparing BAV AS treated with TAVR to SAVR and TAVR for BAV AS versus tricuspid valves. Still, long-term durability data is needed, especially for the newest generation of transcatheter valves. Furthermore, outcomes following surgical explantation of transcatheter aortic valves for structural valve degeneration or progression of aortopathy after TAVR in BAV patients are unknown and may significantly impact lifetime management.

### 1.6. Surgical vs. Transcatheter Replacement for Bicuspid Aortic Valve Stenosis

There is no evidence from an adequately powered RCT to compare TAVR to SAVR in patients with BAV AS. In a systematic review of six retrospective studies, including 6550 patients who underwent aortic valve replacement for BAV AS (3258 TAVR and 3292 SAVR), there were no significant differences between TAVR and SAVR with regards to in-hospital mortality and stroke [42]. Rates of permanent pacemaker implantation and paravalvular leak were higher post-TAVR [33,42,43,44,45,46,47], while rates of acute kidney injury, bleeding requiring blood transfusion, and hospital length of stay were higher following SAVR [42]. The differences in postoperative morbidity are consistent with the existing literature [6,7]. Table 3 summarizes the propensity-matched studies that compared TAVR to SAVR for BAV AS.

There appear to be no significant differences between the two treatment options regarding longer-term mortality, but follow-up is limited. In one study, 1-year mortality was 9.0% in the TAVR cohort and 7.4% in the SAVR cohort [44]. Another report showed a 2-year mortality rate of 9.7% following TAVR and 18.7% following SAVR, with no statistical difference between the groups [43]. More recently, the Nordic Aortic Valve Intervention (NOTION)-2 trial compared TAVR with SAVR in low-risk patients ≤75 years of age, including both tricuspid (270) and BAV (100) AS [49]. The risk of the primary endpoint (a composite risk of death of any cause, stroke, or rehospitalization at 1 year) was numerically higher in the TAVR group compared with SAVR (14.3% vs. 3.9%); however, despite the sizeable absolute risk difference (10.4%), this did not reach statistical significance (hazard ratio [HR] of 3.8, 95% confidence interval [CI], 0.8–18.5; *p* = 0.07). Furthermore, the risk of death or disabling stroke at 1 year was also not statistically different, 6.1% post-TAVR vs. 2.0% post-SAVR. In terms of valve performance, moderate or greater paravalvular regurgitation occurred more frequently in the BAV TAVR cohort as compared to the BAV SAVR cohort (absolute risk difference of 9.1% [95% CI, 0.6%–17.6%], with a three-fold higher risk compared with that observed in the tricuspid cohort. While the totality of the available data suggests at least similar outcomes following TAVR or SAVR in BAV AS, dedicated RCTs comparing TAVR with SAVR in BAV patients are needed.

## 2. Conclusions: Is There Equipoise? Why Randomize?

Only an adequately powered RCT will confirm the optimal treatment strategy for patients with severe BAV AS. TAVR is now a well-established alternative to SAVR for tricuspid anatomy and is supported by the guidelines for patients with less than 20 years to live [13]. But for BAV AS patients, many of whom will present at a much younger age, a TAVR first strategy is not currently supported by evidence. Conversely, in patients with BAV AS and those with concomitant aortopathy, SAVR remains the first-line therapeutic option.

Thus, identifying the appropriate patient population for an RCT in BAV AS patients poses unique questions. Which risk profile would be best suited for such a trial? Should patients with concomitant aortopathy be included or excluded? For one, any RCT dedicated to BAV AS patients would likely involve patients who overlap current indications for both TAVR and SAVR to provide a fair comparison and to allow safe randomization. Current data on TAVR outcomes for BAV AS was derived mainly from highly selected Sievers type 1 patients at intermediate risk of surgery who had relatively stable comorbid conditions without aortopathy meeting current indications for TAVR. Any well-designed RCT should plan for long-term follow-up (minimum of 10 years) to assess mortality, valvular hemodynamics and durability, progression of aortopathy, rehospitalization rates, and other major procedural complications, and thus will require enough young patients with long life expectancy—the same type of patients most would say are best treated with mechanical valves.

**Figure 2:**
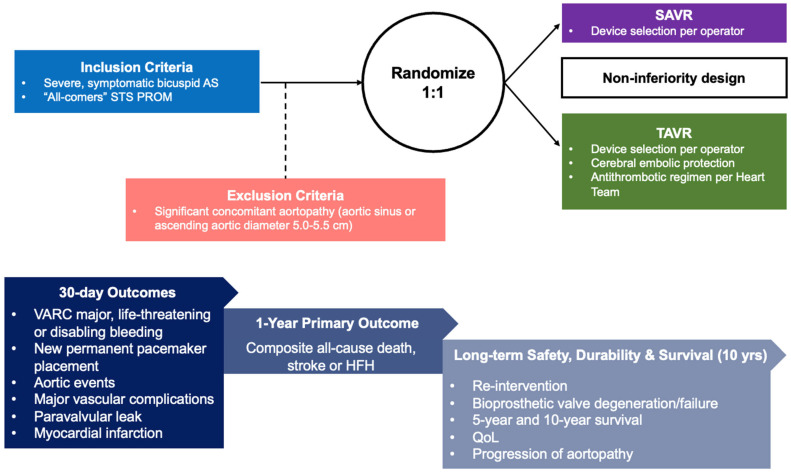
Proposed Study Design. AS: aortic AS; HFH—heart failure hospitalization; SAVR—surgical aortic valve replacement; STS PROM—Society of Thoracic Surgeons Predicted Risk of Mortality; TAVR—transcatheter aortic valve replacement; QoL—quality of life; VARC—valve academic research consortium.

Moreover, for young patients, a Ross procedure at a Valve Center of Excellence offers a return to average life expectancy not seen with any other therapy [50]. Should “any surgical treatment” be compared to TAVR with the valve the heart team believes most appropriate? We hereby offer a hypothetical study design in Figure 2.

Despite the absence of RCTs exploring the outcomes of TAVR in BAV AS, several observational studies and registry data show comparable outcomes for SAVR or TAVR for BAV AS and TAVR in BAV vs. tricuspid AS. Indeed, with continuing advancements in TAVR technology, TAVR may be a practical alternative. But the question remains: Is TAVR feasible in all BAV anatomies? Head-to-head comparisons of SAVR vs. TAVR for BAV AS are required to define the BAV patient subsets most suitable for TAVR. A RCT treatment strategy must enroll young participants, and surgeons and cardiologists alike must believe there is equipoise.

## Figures and Tables

**Table 1:** Comparison of the 2020 ACC/AHA and 2021 ESC/EACTS guidelines for managing bicuspid aortic valve AS with or without concomitant aortic root disease.

2020 ACC/AHA Guidelines [13]	2021 ESC/EACTS Guidelines [17]
TAVR is a potential alternative to SAVR in BAV with severe, symptomatic aortic stenosis (Class 2b)	SAVR is recommended in BAV with severe, symptomatic aortic stenosis
BAV + Aortic sinus or ascending aortic diameter > 5.5 cm, operative replacement of aorta is recommended (Class 1)	Aortic root dilation ≥ 5.5 cm, operative replacement of aorta should be considered (Class 2a)
BAV + Aortic sinus or ascending aortic diameter 5.0–5.5 cm -without symptoms -with additional risk factors * operative replacement is reasonable (Class 2a)	Aortic root disease dilation ≥ 5.0 cm -with additional risk factors * operative replacement of aorta should be considered (Class 2a)
BAV + Aortic sinus or ascending aortic diameter 5.0–5.5 cm -without symptoms -without risk factors operative replacement may be considered (Class 2b)	
**2020 ACC/AHA Guidelines [11]**	**2021 ESC/EACTS Guidelines [12]**
TAVR is a potential alternative to SAVR in BAV with severe, symptomatic aortic AS (Class 2b)	SAVR is recommended in BAV with severe, symptomatic aortic AS
BAV + Aortic sinus or ascending aortic diameter > 5.5 cm, operative replacement of aorta is recommended (Class 1)	Aortic root dilation ≥ 5.5 cm, operative replacement of aorta should be considered (Class 2a)
BAV + Aortic sinus or ascending aortic diameter 5.0–5.5 cm -without symptoms -with additional risk factors * operative replacement is reasonable (Class 2a)	Aortic root disease dilation ≥ 5.0 cm -with additional risk factors * operative replacement of aorta should be considered (Class 2a)
BAV + Aortic sinus or ascending aortic diameter 5.0–5.5 cm -without symptoms -without risk factors operative replacement may be considered (Class 2b)	

TAVR: transcatheter aortic valve replacement; SAVR: surgical aortic valve replacement; BAV: bicuspid aortic valve; AS: aortic stenosis. * Additional risk factors include family history or aortic dissection, aortic growth rate > 0.5 cm per year, and aortic coarctation.

**Table 2:** Characteristics of propensity score matched studies comparing bicuspid to tricuspid aortic AS treated with TAVR.

Author Year	Sample Size	In Hospital Mortality	Stroke	PPI	Bleeding Complication *	Annular or Aortic Dissection	Median Length of Stay (IQR)	30-Day Mortality	1-Year Mortality	2-Year Mortality
Williams 2022 [34]	BAV: 148		2 (1.4)	9 (6.1)				0 (0)	1 (0.7)	
	TAV: 148		2 (1.4)	10 (6.8)				0 (0)	2 (1.4)	
	Matched Total: 296		*p* = 0.62	*p* = 1.0					*p* = 1.0	
Makkar 2021 [32]	BAV: 3168	20 (0.6)	44 (1.4)	189/3035 (6.2)		12 (0.38)		29 (0.9)	146 (4.6)	
	TAV: 3168	13 (0.4)	38 (1.2)	156/3028 (5.2)		9 (0.28)		25 (0.8)	209 (6.6)	
	Matched Total: 6336	*p* = 0.22	*p* = 0.55	*p* = 0.07		*p* = 0.66		*p* = 0.55	*p* = 0.06	
Elbadawi 2019 [33]	BAV: 1035	30 (2.9)	20 (1.9)	145 (14.0)	330 (31.9)		4 (2–7)			
	TAV: 1035	35 (3.4)	20 (1.9)	125 (12.1)	330 (31.9)		4 (2–8)			
	Matched Total: 2070	*p* = 0.76	*p* > 0.999	*p* = 0.51	*p* = 1.0		*p* = 0.07			
Nagaraja 2019 [38]	BAV: 359	20 (5.6)	10 (2.8)	40 (11.1)	123 (34.3)		8.9 ± 9.5			
	TAV: 359	5 (1.4)	20 (5.6)	15 (4.2)	165 (46.0)		8.2 ± 7.6			
	Matched Total: 718		*p* = 0.32		*p* = 0.27		*p* = 0.64			
Yoon 2017 [30]	BAV: 546	7 (1.3)		84 (15.4)		9 (1.6)		20 (3.7)		94 (17.2)
	TAV: 546	6 (1.1)		84 (15.4)		0 (0)		18 (3.3)		106 (19.4)
	Matched Total: 1092	*p* > 0.99		*p* > 0.99				*p* = 0.87		*p* = 0.28

Values are n (%). * Bleeding complication—postoperative bleeding, red blood cell transfusion, reoperation for bleeding, hematoma, or GI bleeding requiring blood transfusion. BAV—bicuspid aortic valve; IQR—interquartile range; PPI—permanent pacemaker implantation; TAV—tricuspid aortic valve; TAVR—transcatheter aortic valve replacement.

**Table 3:** Characteristics of propensity score matched studies comparing TAVR to SAVR for bicuspid aortic valve AS.

Author Year	Sample Size	In Hospital Mortality	Stroke	PPI	Bleeding Complication *	Median Length of Stay (IQR)	30-Day Mortality	1-Year Mortality	2-Year Mortality	Other Follow Up
Chen 2023 [48]										3-year AV reop
	TAVR: 797		19 (2.4)	73 (9.2)		3 (2–6)	13 (1.6)			13 (1.6)
	SAVR: 797		18 (2.3)	50 (6.3)		7 (5–10)	19 (2.4)			14 (1.7)
	Matched Total: 1594		*p* = 0.87	*p* = 0.03		*p* < 0.001	*p* = 0.29			*p* = 0.55
Majmunder 2022 [46]	TAVR: 1393	10 (0.7)	41 (2.9)	155/1309 (11.8)	35 (2.5)	2 (1–4)				
	SAVR: 1393	26 (1.8)	45 (3.2)	113/1314 (8.6)	145 (10.5)	6 (5–8)				
	Matched Total: 3761	*p* = 0.035	*p* = 0.717	*p* = 0.033	*p* < 0.001	*p* < 0.001				
Husso 2021 [43]	TAVR: 75		3 (4.0)	8 (11.4)	11 (14.7)	4.4 ± 3.0	1 (1.3)		(9.7)	
	SAVR: 75		6 (8.0)	4 (5.5)	61 (81.3)	8.1 ± 4.4	4 (5.3)		(18.7)	
	Matched Total: 150		*p* = 0.508	*p* = 0.388	*p* < 0.0001	*p* < 0.0001	*p* = 0.375		*p* = 0.268	
Tsai 2021 [47]	TAVR: 48	6 (12.5)	1 (2.0)			17.3 ± 14.9				
	SAVR: 82	4 (4.9)	0 (0)			17.3 ± 8.2				
	Matched Total ‡: 130	*p* = 0.22	*p* = 0.79							
Mentias 2020 [44]	TAVR: 699	15 (2.2)	19 (2.7)	85 (12.2)	37 (5.3)		20 (2.9)	63 (9.0)		
	SAVR: 699	16 (2.3)	20 (2.9)	53 (7.6)	150 (21.5)		19 (2.7)	52 (7.4)		
	Matched Total: 1398	*p* = 0.90	*p* = 0.90	*p* = 0.009			*p* = 0.90	*p* = 0.34		
Soud 2020 [45] Retro	TAVR: 68	4 (5.9)	1 (1.5)	7 (10.3)	5 (7.4)					
	SAVR: 68	0 (0)	0 (0)	7 (10.3)	9 (13.2)					
	Matched Total: 136	*p* = 0.11	*p* = 1.00	*p* = 1.00	*p* = 0.39					
Elbadawi 2019 [33]	TAVR: 975	30 (3.1)	20 (2.1)	132 (13.8)	300 (30.8)	4 (2–7)				
	SAVR: 975	30 (3.1)	25 (2.6)	45 (4.6)	700 (71.8)	7 (5–9)				
	Matched Total: 1950	>0.999	*p* = 0.55	*p* < 0.001	*p* < 0.001	*p* = 0.01				

Values are n (%). * Bleeding complication—postoperative bleeding, RBC transfusion, reoperation for bleeding, hematoma, or GI bleeding requiring blood transfusion. ‡ These patients were matched 1:2 for age and sex. AV—Aortic valve; IQR—interquartile range; PPI—permanent pacemaker implantation; SAVR—surgical aortic valve replacement; TAVR—transcatheter aortic valve replacement.

## Abbreviations

ACC	American College of Cardiology
AHA	American Heart Association
AS	aortic AS
BAV	bicuspid aortic valve
RCT	randomized controlled trial
SAVR	surgical aortic valve replacement
TAVR	transcatheter aortic valve replacement

## References

[1] Michelena H.I., Della Corte A., Prakash S.K., Milewicz D.M., Evangelista A., Enriquez-Sarano M. (2015). Bicuspid aortic valve aortopathy in adults: Incidence, etiology, and clinical significance. Int. J. Cardiol..

[2] Leon M.B., Smith C.R., Mack M., Miller D.C., Moses J.W., Svensson L.G., Tuzcu E.M., Webb J.G., Fontana G.P., Makkar R.R. (2010). Transcatheter aortic-valve implantation for aortic stenosis in patients who cannot undergo surgery. N. Engl. J. Med..

[3] Smith C.R., Leon M.B., Mack M.J., Miller D.C., Moses J.W., Svensson L.G., Tuzcu E.M., Webb J.G., Fontana G.P., Makkar R.R. (2011). Transcatheter versus surgical aortic-valve replacement in high-risk patients. N. Engl. J. Med..

[4] Leon M.B., Smith C.R., Mack M.J., Makkar R.R., Svensson L.G., Kodali S.K., Thourani V.H., Tuzcu E.M., Miller D.C., Herrmann H.C. (2016). Transcatheter or Surgical Aortic-Valve Replacement in Intermediate-Risk Patients. N. Engl. J. Med..

[5] Reardon M.J., Van Mieghem N.M., Popma J.J., Kleiman N.S., Søndergaard L., Mumtaz M., Adams D.H., Deeb G.M., Maini B., Gada H. (2017). Surgical or Transcatheter Aortic-Valve Replacement in Intermediate-Risk Patients. N. Engl. J. Med..

[6] Mack M.J., Leon M.B., Thourani V.H., Makkar R., Kodali S.K., Russo M., Kapadia S.R., Malaisrie S.C., Cohen D.J., Pibarot P. (2019). Transcatheter Aortic-Valve Replacement with a Balloon-Expandable Valve in Low-Risk Patients. N. Engl. J. Med..

[7] Popma J.J., Deeb G.M., Yakubov S.J., Mumtaz M., Gada H., O’Hair D., Bajwa T., Heiser J.C., Merhi W., Kleiman N.S. (2019). Transcatheter Aortic-Valve Replacement with a Self-Expanding Valve in Low-Risk Patients. N. Engl. J. Med..

[8] Sievers H.H., Schmidtke C. (2007). A classification system for the bicuspid aortic valve from 304 surgical specimens. J. Thorac. Cardiovasc. Surg..

[9] Vincent F., Ternacle J., Denimal T., Shen M., Redfors B., Delhaye C., Simonato M., Debry N., Verdier B., Shahim B. (2021). Transcatheter Aortic Valve Replacement in Bicuspid Aortic Valve Stenosis. Circulation.

[10] Philip F., Faza N.N., Schoenhagen P., Desai M.Y., Tuzcu E.M., Svensson L.G., Kapadia S.R. (2015). Aortic annulus and root characteristics in severe aortic stenosis due to bicuspid aortic valve and tricuspid aortic valves: Implications for transcatheter aortic valve therapies. Catheter. Cardiovasc. Interv..

[11] Michelena H.I., Khanna A.D., Mahoney D., Margaryan E., Topilsky Y., Suri R.M., Eidem B., Edwards W.D., Sundt T.M., Enriquez-Sarano M. (2011). Incidence of aortic complications in patients with bicuspid aortic valves. JAMA.

[12] Tzemos N., Therrien J., Yip J., Thanassoulis G., Tremblay S., Jamorski M.T., Webb G.D., Siu S.C. (2008). Outcomes in adults with bicuspid aortic valves. JAMA.

[13] Otto C.M., Nishimura R.A., Bonow R.O., Carabello B.A., Erwin J.P., Gentile F., Jneid H., Krieger E.V., Mack M., McLeod C. (2021). 2020 ACC/AHA Guideline for the Management of Patients with Valvular Heart Disease: A Report of the American College of Cardiology/American Heart Association Joint Committee on Clinical Practice Guidelines. Circulation.

[14] Czerny M., Grabenwöger M., Berger T., Aboyans V., Della Corte A., Chen E.P., Desai N.D., Dumfarth J., Elefteriades J.A., Etz C.D. (2024). EACTS/STS Guidelines for diagnosing and treating acute and chronic syndromes of the aortic organ. Eur. J. Cardiothorac. Surg..

[15] Mazzolai L., Teixido-Tura G., Lanzi S., Boc V., Bossone E., Brodmann M., Bura-Rivière A., De Backer J., Deglise S., Della Corte A. (2024). 2024 ESC Guidelines for the management of peripheral arterial and aortic diseases. Eur. Heart J..

[16] Isselbacher E.M., Preventza O., Black J.H., Augoustides J.G., Beck A.W., Bolen M.A., Braverman A.C., Bray B.E., Brown-Zimmerman M.M., Chen E.P. (2022). 2022 ACC/AHA Guideline for the Diagnosis and Management of Aortic Disease: A Report of the American Heart Association/American College of Cardiology Joint Committee on Clinical Practice Guidelines. Circulation.

[17] Vahanian A., Beyersdorf F., Praz F., Milojevic M., Baldus S., Bauersachs J., Capodanno D., Conradi L., De Bonis M., De Paulis R. (2022). 2021 ESC/EACTS Guidelines for the management of valvular heart disease. Eur. Heart J..

[18] Goland S., Czer L.S., De Robertis M.A., Mirocha J., Kass R.M., Fontana G.P., Chang W., Trento A. (2007). Risk factors associated with reoperation and mortality in 252 patients after aortic valve replacement for congenitally bicuspid aortic valve disease. Ann. Thorac. Surg..

[19] Borger M.A., David T.E. (2005). Management of the valve and ascending aorta in adults with bicuspid aortic valve disease. Semin. Thorac. Cardiovasc. Surg..

[20] Girdauskas E., Disha K., Raisin H.H., Secknus M.-A., Borger M.A., Kuntze T. (2012). Risk of late aortic events after an isolated aortic valve replacement for bicuspid aortic valve stenosis with concomitant ascending aortic dilation. Eur. J. Cardiothorac. Surg..

[21] Kim Y.G., Sun B.J., Park G.-M., Han S., Kim D.-H., Song J.-M., Kang D.-H., Song J.-K. (2012). Aortopathy and bicuspid aortic valve: Haemodynamic burden is main contributor to aortic dilatation. Heart.

[22] Girdauskas E., Disha K., Borger M.A., Kuntze T. (2014). Long-term prognosis of ascending aortic aneurysm after aortic valve replacement for bicuspid versus tricuspid aortic valve stenosis. J. Thorac. Cardiovasc. Surg..

[23] Wijesinghe N., Ye J., Rodés-Cabau J., Cheung A., Velianou J.L., Natarajan M.K., Dumont E., Nietlispach F., Gurvitch R., Wood D.A. (2010). Transcatheter aortic valve implantation in patients with bicuspid aortic valve stenosis. JACC Cardiovasc. Interv..

[24] Mylotte D., Lefevre T., Søndergaard L., Watanabe Y., Modine T., Dvir D., Bosmans J., Tchetche D., Kornowski R., Sinning J.-M. (2014). Transcatheter aortic valve replacement in bicuspid aortic valve disease. J. Am. Coll. Cardiol..

[25] Takagi H., Hari Y., Kawai N., Kuno T., Ando T. (2019). Meta-analysis of transcatheter aortic valve implantation for bicuspid versus tricuspid aortic valves. J. Cardiol..

[26] Kanjanahattakij N., Horn B., Vutthikraivit W., Biso S.M., Ziccardi M.R., Lu M.L.R., Rattanawong P. (2018). Comparing outcomes after transcatheter aortic valve replacement in patients with stenotic bicuspid and tricuspid aortic valve: A systematic review and meta-analysis. Clin. Cardiol..

[27] Yoon S.H., Kim W.-K., Dhoble A., Pio S.M., Babaliaros V., Jilaihawi H., Pilgrim T., De Backer O., Bleiziffer S., Vincent F. (2020). Bicuspid Aortic Valve Morphology and Outcomes After Transcatheter Aortic Valve Replacement. J. Am. Coll. Cardiol..

[28] Yeats B.B., Yadav P.K., Dasi L.P., Thourani V.H. (2022). Treatment of Bicuspid Aortic Valve Stenosis with TAVR: Filling Knowledge Gaps Towards Reducing Complications. Curr. Cardiol. Rep..

[29] Grubb K.J., Gada H., Mittal S., Nazif T., Rodés-Cabau J., Fraser D.G.W., Lin L., Rovin J.D., Khalil R., Sultan I. (2023). Clinical Impact of Standardized TAVR Technique and Care Pathway: Insights from the Optimize PRO Study. JACC Cardiovasc. Interv..

[30] Yoon S.H., Bleiziffer S., De Backer O., Delgado V., Arai T., Ziegelmueller J., Barbanti M., Sharma R., Perlman G.Y., Khalique O.K. (2017). Outcomes in Transcatheter Aortic Valve Replacement for Bicuspid Versus Tricuspid Aortic Valve Stenosis. J. Am. Coll. Cardiol..

[31] Halim S.A., Edwards F.H., Dai D., Li Z., Mack M.J., Holmes D.R., Tuzcu E.M., Thourani V.H., Harrison J.K., Brennan J.M. (2020). Outcomes of Transcatheter Aortic Valve Replacement in Patients with Bicuspid Aortic Valve Disease: A Report from the Society of Thoracic Surgeons/American College of Cardiology Transcatheter Valve Therapy Registry. Circulation.

[32] Makkar R.R., Yoon S.-H., Chakravarty T., Kapadia S.R., Krishnaswamy A., Shah P.B., Kaneko T., Skipper E.R., Rinaldi M., Babaliaros V. (2021). Association Between Transcatheter Aortic Valve Replacement for Bicuspid vs Tricuspid Aortic Stenosis and Mortality or Stroke Among Patients at Low Surgical Risk. JAMA.

[33] Elbadawi A., Saad M., Elgendy I.Y., Barssoum K., Omer M.A., Soliman A., Almahmoud M.F., Ogunbayo G.O., Mentias A., Gilani S. (2019). Temporal Trends and Outcomes of Transcatheter Versus Surgical Aortic Valve Replacement for Bicuspid Aortic Valve Stenosis. JACC Cardiovasc. Interv..

[34] Williams M.R., Jilaihawi H., Makkar R., O’neill W.W., Guyton R., Malaisrie S.C., Brown D.L., Blanke P., Leipsic J.A., Pibarot P. (2022). The PARTNER 3 Bicuspid Registry for Transcatheter Aortic Valve Replacement in Low-Surgical-Risk Patients. JACC Cardiovasc. Interv..

[35] Forrest J.K., Ramlawi B., Deeb G.M., Zahr F., Song H.K., Kleiman N.S., Chetcuti S.J., Michelena H.I., Mangi A.A., Skiles J.A. (2021). Transcatheter Aortic Valve Replacement in Low-risk Patients with Bicuspid Aortic Valve Stenosis. JAMA Cardiol..

[36] Waksman R., Craig P.E., Torguson R., Asch F.M., Weissman G., Ruiz D., Gordon P., Ehsan A., Parikh P., Bilfinger T. (2020). Transcatheter Aortic Valve Replacement in Low-Risk Patients with Symptomatic Severe Bicuspid Aortic Valve Stenosis. JACC Cardiovasc. Interv..

[37] Tchetche D., Ziviello F., De Biase C., De Backer O., Hovasse T., Leroux L., Petronio A.-S., Saint-Etienne C., Teles R.C., Modine T. (2023). Transcatheter aortic valve implantation with the Evolut platform for bicuspid aortic valve stenosis: The international, multicentre, prospective BIVOLUTX registry. EuroIntervention.

[38] Nagaraja V., Suh W., Fischman D.L., Banning A., Martinez S.C., Potts J., Kwok C.S., Ratib K., Nolan J., Bagur R. (2019). Transcatheter aortic valve replacement outcomes in bicuspid compared to trileaflet aortic valves. Cardiovasc. Revasc. Med..

[39] Généreux P., Piazza N., Alu M.C., Nazif T., Hahn R.T., Pibarot P., Bax J.J., Leipsic J.A., Blanke P., VARC-3 WRITING COMMITTEE (2021). Valve Academic Research Consortium 3: Updated Endpoint Definitions for Aortic Valve Clinical Research. J. Am. Coll. Cardiol..

[40] Pibarot P., Herrmann H.C., Wu C., Hahn R.T., Otto C.M., Abbas A.E., Chambers J., Dweck M.R., Leipsic J.A., Simonato M. (2022). Standardized Definitions for Bioprosthetic Valve Dysfunction Following Aortic or Mitral Valve Replacement: JACC State-of-the-Art Review. J. Am. Coll. Cardiol..

[41] Tchetche D., de Biase C., van Gils L., Parma R., Ochala A., Lefevre T., Hovasse T., De Backer O., Sondergaard L., Bleiziffer S. (2019). Bicuspid Aortic Valve Anatomy and Relationship with Devices: The BAVARD Multicenter Registry. Circ. Cardiovasc. Interv..

[42] Kang J.J., Fialka N.M., El-Andari R., Watkins A., Hong Y., Mathew A., Bozso S.J., Nagendran J. (2023). Surgical vs transcatheter aortic valve replacement in bicuspid aortic valve stenosis: A systematic review and meta-analysis. Trends Cardiovasc. Med..

[43] Husso A., Airaksinen J., Juvonen T., Laine M., Dahlbacka S., Virtanen M., Niemelä M., Mäkikallio T., Savontaus M., Eskola M. (2021). Transcatheter and surgical aortic valve replacement in patients with bicuspid aortic valve. Clin. Res. Cardiol..

[44] Mentias A., Sarrazin M.V., Desai M.Y., Saad M., Horwitz P.A., Kapadia S., Girotra S. (2020). Transcatheter Versus Surgical Aortic Valve Replacement in Patients with Bicuspid Aortic Valve Stenosis. J. Am. Coll. Cardiol..

[45] Soud M., Soud M., Al-Khadra Y., Darmoch F., Pacha H.M., Fanari Z. (2020). Transcatheter aortic valve replacement in patients with bicuspid aortic valve stenosis: National trends and in-hospital outcomes. Avicenna J. Med..

[46] Majmundar M., Kumar A., Doshi R., Shariff M., Krishnaswamy A., Reed G.W., Brockett J., Lahorra J.L., Svensson L.S., Puri R. (2022). Early outcomes of transcatheter versus surgical aortic valve implantation in patients with bicuspid aortic valve stenosis. EuroIntervention.

[47] Tsai H.Y., Lin Y., Wu I., Kuo L., Chen B., Shen S., Hsu W., Huang H. (2021). Major adverse cardiac events and functional capacity in patients at intermediate risk undergoing transcatheter versus surgical aortic valve replacement for aortic stenosis with bicuspid valves. J. Card. Surg..

[48] Chen A., Malas J., Megna D., Tam D.Y., Gill G., Rowe G., Premananthan S., Krishnan A., Peiris A., Emerson D. (2024). Bicuspid aortic stenosis: National three-year outcomes of transcatheter versus surgical aortic valve replacement among Medicare beneficiaries. J. Thorac. Cardiovasc. Surg..

[49] Jorgensen T.H., Thyregod H.G.H., Savontaus M., Willemen Y., Bleie Ø., Tang M., Niemela M., Angerås O., Gudmundsdóttir I.J., Sartipy U. (2024). Transcatheter Aortic Valve Implantation in Low-Risk Tricuspid or Bicuspid Aortic Stenosis: The NOTION-2 Trial. Eur. Heart J..

[50] Mazine A., El-Hamamsy I., Verma S., Peterson M.D., Bonow R.O., Yacoub M.H., David T.E., Bhatt D.L. (2018). Ross Procedure in Adults for Cardiologists and Cardiac Surgeons: JACC State-of-the-Art Review. J. Am. Coll. Cardiol..

